# Codesigned Shared Decision-Making Diabetes Management Plan Tool for Adolescents With Type 1 Diabetes Mellitus and Their Parents: Prototype Development and Pilot Test

**DOI:** 10.2196/jopm.9652

**Published:** 2018-05-10

**Authors:** Tamara S Hannon, Courtney M Moore, Erika R Cheng, Dustin O Lynch, Lisa G Yazel-Smith, Gina EM Claxton, Aaron E Carroll, Sarah E Wiehe

**Affiliations:** ^1^ Pediatric and Adolescent Comparative Effectiveness Research Department of Pediatrics Indiana University School of Medicine Indianapolis, IN United States; ^2^ Children's Health Services Research Center Department of Pediatrics Indiana University School of Medicine Indianapolis, IN United States; ^3^ Community Health Partnerships Patient Engagement Core Indiana Clinical and Translational Sciences Institute Indiana University Indianapolis, IN United States

**Keywords:** adolescent health services, patient-centered care, research design, diabetes mellitus, type 1, self-management

## Abstract

**Background:**

Adolescents with type 1 diabetes mellitus have difficulty achieving optimal glycemic control, partly due to competing priorities that interfere with diabetes self-care. Often, significant diabetes-related family conflict occurs, and adolescents’ thoughts and feelings about diabetes management may be disregarded. Patient-centered diabetes outcomes may be better when adolescents feel engaged in the decision-making process.

**Objective:**

The objective of our study was to codesign a clinic intervention using shared decision making for addressing diabetes self-care with an adolescent patient and parent advisory board.

**Methods:**

The patient and parent advisory board consisted of 6 adolescents (teens) between the ages 12 and 18 years with type 1 diabetes mellitus and their parents recruited through our institution’s Pediatric Diabetes Program. Teens and parents provided informed consent and participated in 1 or both of 2 patient and parent advisory board sessions, lasting 3 to 4 hours each. Session 1 topics were (1) patient-centered outcomes related to quality of life, parent-teen shared diabetes management, and shared family experiences; and (2) implementation and acceptability of a patient-centered diabetes care plan intervention where shared decision making was used. We analyzed audio recordings, notes, and other materials to identify and extract ideas relevant to the development of a patient-centered diabetes management plan. These data were visually coded into similar themes. We used the information to develop a prototype for a diabetes management plan tool that we pilot tested during session 2.

**Results:**

Session 1 identified 6 principal patient-centered quality-of-life measurement domains: stress, fear and worry, mealtime struggles, assumptions and judgments, feeling abnormal, and conflict. We determined 2 objectives to be principally important for a diabetes management plan intervention: (1) focusing the intervention on diabetes distress and conflict resolution strategies, and (2) working toward a verbalized common goal. In session 2, we created the diabetes management plan tool according to these findings and will use it in a clinical trial with the aim of assisting with patient-centered goal setting.

**Conclusions:**

Patients with type 1 diabetes mellitus can be effectively engaged and involved in patient-centered research design. Teens with type 1 diabetes mellitus prioritize reducing family conflict and fitting into their social milieu over health outcomes at this time in their lives. It is important to acknowledge this when designing interventions to improve health outcomes in teens with type 1 diabetes mellitus.

## Introduction

Type 1 diabetes mellitus (T1DM) is diagnosed in approximately 1 in 400 US youth under the age of 20 years, making it one of the most common childhood chronic diseases [1]. Adolescents with T1DM have significant difficulty achieving optimal glycemic control due to challenges in shifting and evolving social priorities that can interfere with medication adherence, increasing insulin requirements characteristic of puberty, diabetes-related distress, and family conflict [2-4]. A principal challenge of intensive diabetes care is maintaining frequent self-monitoring of blood glucose (SMBG) and insulin dosing. A clinical strategy to increase adherence to medical recommendations is real-time sharing of adolescent SMBG or continuous glucose monitor data with parents. Health information technology (HIT) allows real-time sharing of SMBG and messaging between patient, parents, and health care providers (ie, HIT-enhanced SMBG). HIT-enhanced SMBG has been shown to improve reactive and proactive blood glucose management, provide adherence support, and promote intensification of treatment [5-7]. However, adolescents and parents are often reluctant to adopt this technology, which may be related to parental nagging, family conflict, and additional burden or stress placed on adolescents and parents [3,4,8-12].

The general well-being of patients and parents is significantly affected by the demands of daily diabetes care, the even lower glycemic control targets, and the monetary costs of diabetes therapies [13,14]. It is not surprising that patients, parents, and diabetes care providers can have conflicting ideas about optimal treatments and therapeutic goals, as some treatments may further increase patient burden, affecting clinical and psychosocial outcomes. For example, a health care provider may want the patient to use a newer technology for SMBG to improve glycemic control, but the patient may feel that this will further increase his or her stress levels, and stress may be the primary outcome of importance to them at this time. A patient-centered approach using shared decision making to identify self-care goals during the clinical encounter could reduce diabetes distress and improve diabetes self-care among adolescents with T1DM using HIT-enhanced SMBG. Shared decision making in person-centered care is a process in which clinicians and patients work together to make decisions and select care plans based on clinical evidence that balances risks and expected outcomes with patient preferences and values [15]. Diabetes self-care goals outlined with the diabetes care team in the form of behavioral contracts have been used to address (1) goals for the frequency of SMBG, (2) goals for the frequency of contact with the diabetes clinic team, and (3) parent and youth responsibilities [6,16-18]. However, these contracts can be perceived as punitive if they are not constructed using patient-centered communication, as adolescents can be sensitive to authoritarian treatment [19].

In this study we collaborated with a patient and parent advisory board (PAB) to (1) outline major causes of diabetes-related distress affecting quality of life; (2) identify the patient-centered health outcomes most important to the PAB participants; and (3) determine how to incorporate shared decision making in the clinic setting when a health care provider, a patient, and a parent may have different goals. The principal objective was to codesign, with the PAB, an intervention that used shared decision making in the creation of a diabetes management plan. We would then test the resulting clinic intervention in a future study of adolescents with T1DM using HIT-enhanced SMBG (NCT02115555). We hypothesized that a PAB-codesigned clinic intervention would prioritize outcomes that differed from routinely measured and highly emphasized medical outcomes. Here, we describe the strategy for working with a PAB on this project and the development of a PAB-codesigned shared decision-making tool for use with adolescents with T1DM and their parents.

## Methods

### Participants

To codesign the shared decision-making strategy, we first formed a PAB that consisted of adolescents with T1DM and their parents. Inclusion criteria required youth to be between 12 and 18 years of age, have T1DM diagnosed for at least 6 months, be a patient in our Pediatric Diabetes Program at the Indiana University School of Medicine, Indianapolis, IN, USA, and have a parent or guardian who agreed to participate. To convene the PAB, we invited adolescents (teens) between the ages 12 and 18 years with T1DM who were seen in our Pediatric Diabetes Program clinical practice in the past 3 months and their parent(s) to be advisors. The goals of the PAB were to allow for (1) active engagement between scientists and patients, (2) a partnership in designing the shared decision-making strategy, and (3) development of the implementation strategy for the funded randomized controlled trial. Teens and parents provided informed consent and participated in 1 or both of 2 PAB sessions, each lasting 3 to 4 hours.

To accomplish our study aims, we partnered with the Indiana Clinical and Translational Sciences Institute Patient Engagement Core (PEC), a team of human-centered designers that offers services to academic researchers related to patient-centered outcome measurement, recruitment, and study acceptability. With this approach, researcher and research participant hold parity and shared inquiry, and designers serve as translators to bridge the communication gap between researchers and patients. We used a systems design approach that could engage participants in the design thinking process. This would take participants through the stages of defining the problems and barriers, generating ideas and solutions, and prototyping an approach or tool to address a problem or barrier. Similar methods have been used in development approaches to address self-management of type 2 diabetes, pediatric asthma, and quality improvement health care facilities [20-22]. The methods employed by the PEC are highly interactive and leverage the expertise of research participants in ways that exceed standard expectations for study participation [23-26]. By combining qualitative research methods with novel methods from design research, the PEC is able to create truly innovative approaches for the engagement of patients and caregivers in research [27-29]. This partnership allowed us to fully engage participants in the development process and provided an opportunity for PAB members to be open about their experiences without fear of judgment or reproach from physician stakeholders.

We managed study data using Research Electronic Data Capture (REDCap) tools hosted at the Indiana Clinical and Translational Sciences Institute and at the Indiana University Pervasive Technology Institute [30], which supports REDCap with information technology infrastructure and consulting resources.

### Conducting the Type 1 Diabetes Patient and Parent Advisory Board Meetings

The PEC facilitated 2 sessions with the PAB that were designed to identify patient-centered outcomes important to the participants and recommendations for how to incorporate shared decision making to create a diabetes self-care contract in the clinic setting. Each session consisted of a variety of group activities designed to break down barriers and inhibitions to verbal participation, promote rapport, and engage participants. For the first session, the PEC designed activities to (1) elicit patient-centered outcomes related to quality of life, parent-teen shared diabetes management, and shared family experiences; and (2) facilitate discussion regarding the implementation and acceptability of the proposed intervention (self-care contract). The PEC used this information to develop a prototype for a diabetes management plan tool to be used in the clinic setting, which would guide shared decision making [27]. Acceptability and clinical implementation of this prototype were the focus of the second session. Table 1 presents participant characteristics and session objectives and activities. Session 1 was attended by 12 patient advisors (6 teens and 6 parents). Session 2 took place approximately 2 months after session 1 and was attended by 6 patient advisors (3 teens and 3 parents). Unfortunately, some participants were lost for session 2 due to scheduling difficulties.

#### Session 1

To assess important patient-centered outcomes, we asked participants to write their response to the question “How does diabetes most impact your life?” on a notecard. A PEC team member then read each response aloud to the entire group and asked them to guess whether the response was written by a parent or a teen. The purpose of this activity was to assess the extent to which diabetes affects parent and teen quality of life, while also uncovering any impacts of diabetes that are shared between teens and parents. A follow-up discussion followed to examine each quality-of-life impact shared by the teen and parent in more detail.

Because diabetes self-care in adolescents is often affected by conflict with parents, we considered the possibility that family conflict might be an important patient-centered outcome. A separate activity asked participants to reflect on aspects of diabetes management that cause conflict within their families. For this activity, we placed teens and parents in separate rooms and recorded their responses on flipchart paper. We then exchanged these responses, and we asked the teens to suggest solutions to the conflicts that the parents wrote, and asked the parents to suggest solutions to the conflicts the teens wrote.

Using standard diabetes self-management tasks as starting goals, and the feedback on how diabetes distress and family conflict affect the completion of these tasks that we collected from these discussions, PEC design team members (CMM and DOL) then developed a prototype for a diabetes management plan tool. The purpose of this tool was to guide shared decision making between teens and parents to establish patient-centered goals, propose diabetes self-care actions, and create behavioral rewards for both teens and parents.

#### Session 2

During the second session, we reconvened the PAB to discuss and pilot test the diabetes management plan tool prototype. The purpose of this session was to uncover any problems with the diabetes management plan tool process, its content, and its acceptability within individual and family contexts. In this session, we asked each parent-teen dyad to complete activities contained in the tool and to provide feedback. The PEC facilitators observed this activity and recorded notes; they did not assist with the process. Teens and parents provided feedback together and as separate groups. After meeting separately, the entire group reconvened to share the main points of their independent discussions. We also asked them to brainstorm solutions when issues with the prototype were identified (eg, readability, functionality, fit, challenges, or perceived value) and to discuss what should happen if future users of the tool were not willing to complete the activities.

**Table 1:** Patient and parent advisory board meeting objectives and activities.

Session	Participants^a^	Objectives	Activities
1	6 teens (4 male, median age 14.6, range 12.4-16.4 years), 6 parents	Patient-centered outcomes; negotiation tactics	Participant-generated card sorting; role reversal
2	3 teens (3 male, median age 14.7, range 14.0-16.6 years), 3 parents	Prototype testing; prototype refinement	Role play; observation; cognitive interview

^a^Demographic information such as race or date of diagnosis was not collected from participants.

### Analysis

We based analyses for PAB-derived activities on Ackoff’s data, information, knowledge, and wisdom scheme, which structures data collection and analysis in a manner that culminates in theory (explanations of human problems) and concept development (creation of new ways to handle problems) [31]. We used an inductive descriptive approach and thematic analysis [32,33]. This framework is applied in settings where computer-aided decision making is used, including informatics [34,35], but it is also used in design research [36]. This process evolves across 4 categories of interpretation: data (eg, written, audio, or video review), information (eg, items of importance or significance written on sticky notes), knowledge (eg, finding patterns to identify themes and areas of importance), and wisdom (eg, applying knowledge to create something new or to make decisions).

Using this framework, the PEC reviewed audio recordings and detailed notes from session 1 (data). They then analyzed notes and other materials generated during the sessions to identify and extract key ideas participants expressed that were relevant to the development of a diabetes care plan (information). These ideas were written onto separate sticky notes and then visually coded into similar themes (knowledge). Some of these themes dealt with domains of agreement terms to be included in the plan, and others dealt with the ideal use of such a plan and ideal interactions around its use. For each of the patient-centered diabetes themes (domains), we identified previously validated questionnaires, if possible, that addressed the corresponding patient-centered outcomes. We did not administer these questionnaires in this study, but they could be used in future outcomes research to assess diabetes distress.

The PEC investigators used the knowledge gained through gathering and analyzing session 1 data, as well as existing disciplinary knowledge (visual communication and design expertise) to create a tool to be tested in session 2. The prototype tool used in session 2 is the initial application of this knowledge (demonstrating wisdom). We then tested the tool and analyzed the resulting data using a deductive approach with specific domains of desired feedback determined ahead of the session. We analyzed these new data in the same fashion as above to identify new knowledge that further refined the developed wisdom.

## Results

### Session 1: Patient-Centered Outcomes

Using the “How does diabetes most impact your life?” notecards from session 1 and the ensuing discussion themes, we identified 6 principal patient-centered quality-of-life measurement domains affecting parents and teens. For each of these domains, we report representative quotes below. Table 2 shows these patient-centered domains, along with validated questionnaires that could be used to address these domains and diabetes distress in future outcomes research.

#### Stress

The theme of stress was the most common theme expressed by teens. Teens were stressed about whether they had all of the supplies they needed, remembering all of the tasks they were asked to perform, and fitting the additional requirements of diabetes self-care into their busy lives while still fitting in with peers. Some of these feelings are summarized by the following quotes:

Diabetes affects me by putting a lot of stress on me.

Teen participant

Diabetes doesn’t limit my life, but it is a daily thing...I do worry every day about my health, even though I know how to take care of myself.

Teen participant

Yeah at my school, I’m the only diabetic...and the teachers hardly know what to do. There’s no school nurse there either. So it’s hard for me. I’m having to deal with school, homework, the sports I’m playing, and also my diabetes.

Teen participant

#### Fear and Worry

The theme of fear and worry related to diabetes was pervasive in nearly every aspect of the parents’ daily lives. Of the 12 impact notecards, 7 included something about worry, stress, or fear. In contrast to teens, parents expressed concern about potential worst-case outcomes (eg, nighttime hypoglycemia), preparing their children for life on their own, and balancing giving their children freedom while keeping them safe. The following quotes illustrate the fear parents expressed feeling:

I just think all the parents locked in on the word fear. I think the difference between [fear for the child and parent is] the parents are programmed to be concerned for the kids. So, yeah we’re all afraid for them and we all have their best interests at heart. The kids, on the other hand, I wonder if they realize how pernicious the stuff can be and what they’re most concerned about is, “Don’t label me. I want to be like everybody else. Let me live my life.” And somewhere those have to meet for some success. And, you know, I was a teenager. Rules, to a certain extent, are meant to be broken I guess. It’s how we, sort of, test the limit and how we grow. But I don’t think we can afford that latitude here, which is why fear has a bigger heartfelt meaning for most of the parents.

Parent participant

I am afraid of the future for my child and afraid of nighttime lows that I won’t be able to wake him up from.

Parent participant

**Table 2:** Quality-of-life measurement domains and pertinent diabetes distress outcomes measures.

Domain and desired outcomes		Study outcomes measures (questionnaires)
**Stress**		
	Diabetes-related stress reduced for teens	DAWN Problem Areas in Diabetes Questionnaire^a^; Peds Quality of Life Inventory Diabetes Module
**Fear and worry**		
	Diabetes-related stress reduced	DAWN Problem Areas in Diabetes Questionnaire; Parental Environment Questionnaire Peds Quality of Life Inventory Diabetes Module
	Teens more effective at managing diabetes	Child Adherence in Diabetes Questionnaire; Laboratory results (hemoglobin A_1c_)
	Teen to manage diabetes independently at times	Parental Environment Questionnaire; Child Diabetes Family Conflict Scale
	Communicate productively about fear and worry	Child Adherence in Diabetes Questionnaire; Parental Environment Questionnaire; Child Diabetes Family Conflict Scale
**Mealtime**		
	Mealtime isn’t overly burdensome	DAWN Problem Areas in Diabetes Questionnaire; Child Adherence in Diabetes Questionnaire
	Teen feels involved in activities and celebrations	DAWN Problem Areas in Diabetes Questionnaire
**Assumptions and judgments**		
	Effectively communicate realities of diabetes	DAWN Problem Areas in Diabetes Questionnaire; Peds Quality of Life Inventory Diabetes Module
	Skills to manage judgment and bullying	N/A^b^
	Skills to advocate for needed support	DAWN Problem Areas in Diabetes Questionnaire; Peds Quality of Life Inventory Diabetes Module
	Teens feel understood and accepted	DAWN Problem Areas in Diabetes Questionnaire
**Normalcy or fitting in**		
	Teen feels involved with peers	DAWN Problem Areas in Diabetes Questionnaire; Patient Health Questionnaire^c^ (PHQ-9)
	Teen advocates for being treated as equal	DAWN Problem Areas in Diabetes Questionnaire; Peds Quality of Life Inventory Diabetes Module
	Teen can express individual symptoms and needs	DAWN Problem Areas in Diabetes Questionnaire; Patient Health Questionnaire (PHQ-9)
	Teen can take part in extracurricular activities	DAWN Problem Areas in Diabetes Questionnaire
	Parent doesn’t assume that expressions of emotion are diabetes related	N/A
	Parents have similar rules for teens with and without type 1 diabetes	N/A
**Conflict**		
	Teens and parents resolve disputes productively	Parental Environment Questionnaire; Child Diabetes Family Conflict Scale
	Parents manage conflicts in consistent fashion	N/A
	Parents don’t yell, take frustrations out on teen	Parental Environment Questionnaire
	Teen is honest about self-monitoring of blood glucose and self-care	N/A
	Teen is given a chance to explain himself or herself	N/A

^a^DAWN Problem Areas in Diabetes Questionnaire has both a pediatric and a parent version.

^b^N/A: not applicable (available questionnaires lack the ability to assess competence in this area; further questions are needed).

^c^Assessment of depressive symptoms.

In addition, teens and parents discussed that many schools did not have the resources to properly care for their children during the school day and that many people, including teachers and coaches, did not seem to understand the seriousness of acute and chronic management of T1DM. This was both a source of major concern for parents because it made their children vulnerable (amplifying their fear) and a source of frustration for teens when school staff minimized the daily struggles they face. Here are some representative quotes from parents:

When he was in junior high, there wasn’t a nurse and there were two nurses for the entire school system and they go to each school like one day a week...Being worried all the time while he was at school, not knowing who was going to be taking care of him and that caused stress at my job. I was a manager at that time. I had a lot of phone calls back and forth throughout the day when the nurse wasn’t there. He would drop really low, go really high, it was all over the place. It got to the point that it was enough stress at work that I was told I was either a manager or the mother of a diabetic. I stepped down.

Parent participant

My biggest concern is that our coaches in sports he’s getting ready to play in high school are not going to take him seriously or they’re going to be—pull him out when he looks bad. I don’t want them to do that. I want him to know when he’s low or when he’s high instead of somebody else looking at him and saying, “Well he’s diabetic, let’s just...”

Parent participant

#### Mealtime Struggles

Mealtime struggles affecting the entire family were shared by parents and teens. Teens also reported feeling hungry but not being able to eat for reasons such as blood sugar levels not being in range, not wanting to eat certain foods that were available, or being full but having to finish their meal because they had already dosed insulin for it. Some of these struggles are represented by the following quotes:

You’re growing and you get hungrier...You can’t [eat] like normal people do. They can just eat whatever they want when they want. For us it’s kind of harder because you can only eat so much at a time.

Teen participant

You put your insulin in before you eat and maybe you get full but you can’t take that insulin back. So you gotta force it down.

Teen participant

Teens and parents specifically mentioned difficulties faced during holidays because many traditions are focused on food and, in many cases such as Valentine’s Day and Halloween, focus on high-sugar foods. As expressed by the following quotes, an overabundance of sweets and food can be bothersome:

Holidays and Halloween and Valentine’s Day are centered around candy and you just can’t pop the 10 candy bars in your mouth like you used to be able to do.

Teen participant

Every holiday we have, we celebrate with food. We always have food...When you have to take insulin for it, you think about it more. We have food around us a lot.

Parent participant

Parents discussed how their child’s diabetes dictates where families can go out to eat and what they can eat at home:

The one most impacting thing is EATING! It impacts the entire family and extended family. There are so many aspects of meals—timing, what we are eating, when, where, what...etc.

Parent participant

If she’s very high and she wants to eat—you’re 300, you’re not going to eat right now. You need to wait until your blood sugar is down.

Parent participant

#### Assumptions and Judgments

The theme of assumptions and judgments included those felt by the teen or parent from others and assumptions that parents made when relating to their child with diabetes. Teens and parents discussed several points of frustration caused by assumptions and judgments that others placed on them or their child. These included misunderstandings about the difference between type 1 and type 2 diabetes and poor understanding about T1DM. Teens discussed facing criticism when they ate sugar, confusion from their peers about why they were not overweight, and stares when they performed SMBG and dosed insulin in public. The following quotes represent the feelings of teens, who sometimes felt ashamed to put their diabetes “on display:”

Diabetes has really impacted the way people judge what I can and can’t do, and normally they don’t know, they just assume. Most people assume I can’t eat anything sweet or when I’m low, people judge me for that.

Teen participant

I remember...I told someone I had diabetes and they were like, “Eww get away from me” because he actually did think I was contagious and it was just the most awkward thing.

Teen participant

When I check my sugar [in class], I get stared at the entire time I do it and it’s just extremely embarrassing. I just wanna leave. Every part of me is telling me to leave. I don’t want them staring at me...I don’t really want to have to do it in front of everyone.

Teen participant

The first couple years, my dad said that I had to go to the car...my mom got so mad if I [checked my blood sugar] in public. Indecency she thought. She’s lightened up. That’s why I do it in secrecy. It kind of rubbed off. I like go in my backpack because I try to be as discreet as possible.

Teen participant

Parents found themselves assuming that every mood swing is blood sugar related. This assumption frustrated teens, as they wanted to be able to express emotions without being tied to their T1DM. Parents recognized this, as exemplified in the quotes below:

I think I attribute a lot of things that probably have nothing to do with it. He’ll do something and I’ll [question him] and he’ll be like, “No.” That’s the first thing I go to and I feel bad for that.

Parent participant

It’s hard for me because we’ll be joking around being silly and all of a sudden I’ll be like, “Is he low?” and then he gets mad at me like, “can’t I ever have fun without you thinking the worst?”

Parent participant

#### Feeling Abnormal

The theme of feeling abnormal was a major concern for teens. Teens discussed many ways in which diabetes prevented them from of having a sense of normalcy. As one teen put it:

I feel like I’m special needs or something.

Teen participant

Some teens and parents described how teens with diabetes were sometimes separated from peers during important activities in school, such as for testing. For some, this was seen as helpful because it allowed for some leeway in the event of diabetes complications, but for others, this separation was perceived as singling them out negatively. Some of the parents’ thoughts on this are represented here:

We have a plan written to where...if he has a high or low during a test like that he can retake the test...but they’ve never taken him aside. They keep him with everybody else.

Parent participant

I found out a while back...when it was test time, they would take all of the type 1 kids and put them in a specific location. So it’s almost a second-class setup—even if you perceive it as a benefit, which it is because they can monitor and see if they’re low and stuff like that—the downside is, what do teenagers want? To be like everybody else.

Parent participant

Parents were also concerned about relating to their teen as numbers instead of as an individual person because of their focus on diabetes management. Parents also expressed their struggles to find a balance between keeping their children safe and allowing them the freedom to be a “normal” teen. Parents wanted their children to spend time with friends but were frustrated and worried when their teens neglected to perform diabetes self-care while at friends’ houses. Their thoughts represented the internal struggle they had when their child with diabetes was away from them:

I won’t let her just go to the mall or anywhere unless I know specifics or an adult is going to be there. Whereas before I would let her and a friend play outside for hours or go on a bike ride. I can’t let her go on a bike ride. I need to make sure if she goes low someone is there. It’s any activity that’s out of your eyesight.

Parent participant

I have to know the parents well and I have to know the parents can take care of him before I [let my child spend time with them]. And he wants to go spend the night with who he wants to go spend the night with. There’s a conflict.

Parent participant

They feel like they can handle this. And they don’t understand that when they’re low, they can’t handle this.

Parent participant

In addition, many parents reported feeling unsure of how they would be able to allow their teens with T1DM to have the same rules as older siblings and teens. These quotes highlight this struggle:

I think dating is going to be a real obstacle when it comes but when it does that’ll be a different thing completely than what my brothers went through because with my brothers, they are just like “Okay have fun” but with me it’ll be like “Make sure you do this, make sure you do this, make sure you do this.” And then they’re going to inform whoever I’m going out with, “Hey if he’s doing this, do that, do that.”

Teen participant

I always think about my 18-year-old daughter and the things she’s able to do. Driving, of course, or going on Spring Break or she’s been on some mission trips. And I worry about how am I going to let her do those things too and am I going to and...

Parent participant

#### Conflict

The theme of conflict was important for teens and parents alike. The PAB discussed conflict resolution between parents and teens, specifically in the context of 3 other domains identified in the session: stress, fear, and not being able to live like a “normal” teen. Both parents and teens cited conflict resolution as an important outcome. The main concern for teens was dreading having to tell parents when they have high blood sugar numbers because they did not want to be yelled at or questioned. They expressed a desire to explain themselves. Many of the teens had a parent they preferred to tell because the reaction was more desirable. Some examples of how teens feel about conflict over blood sugars are here:

When I have a high number, I’d much rather tell my mom—she’s really scary too—but my dad’s like scarier because...If I tell my dad I’m like 170 or something, he’ll be like, “What’s wrong with you? What did you eat?” and I was like, “I didn’t do anything.” My mom would be like, “Oh my god, you are in trouble” and I’ll be like “Don’t tell my dad.” And she’ll keep it low key but my dad will be like yelling at me.

Teen participant

I like to talk to my dad more about numbers and stuff because he’s just more easygoing...my mom will yell at me and I don’t think that really gets anywhere, yelling at me. Sometimes it’s not my fault.

Teen participant

Teens understood and accepted parental concerns and associated parental behaviors. In turn, parents understood that their children must be able to manage their illness independently. Both parents and teens desired a better system for conflict resolution and better skills for working together.

### Session 1: Acceptability of Intervention

None of the dyads in the PAB had an official written agreement related to diabetes management. The consensus in the group was that parents and teens tend to have an unspoken understanding wherein teens knew when to notify parents of blood sugar levels and that parents would review their children’s SMBG. Both parents and teens in the PAB understood and acknowledged the importance of comanagement in diabetes self-care. The group consensus was that 2 objectives were principally important: (1) focusing the intervention on conflict resolution strategies, and (2) working toward a common goal (Table 3).

### Session 2: Diabetes Management Plan Tool Prototype

**Figure 1:**
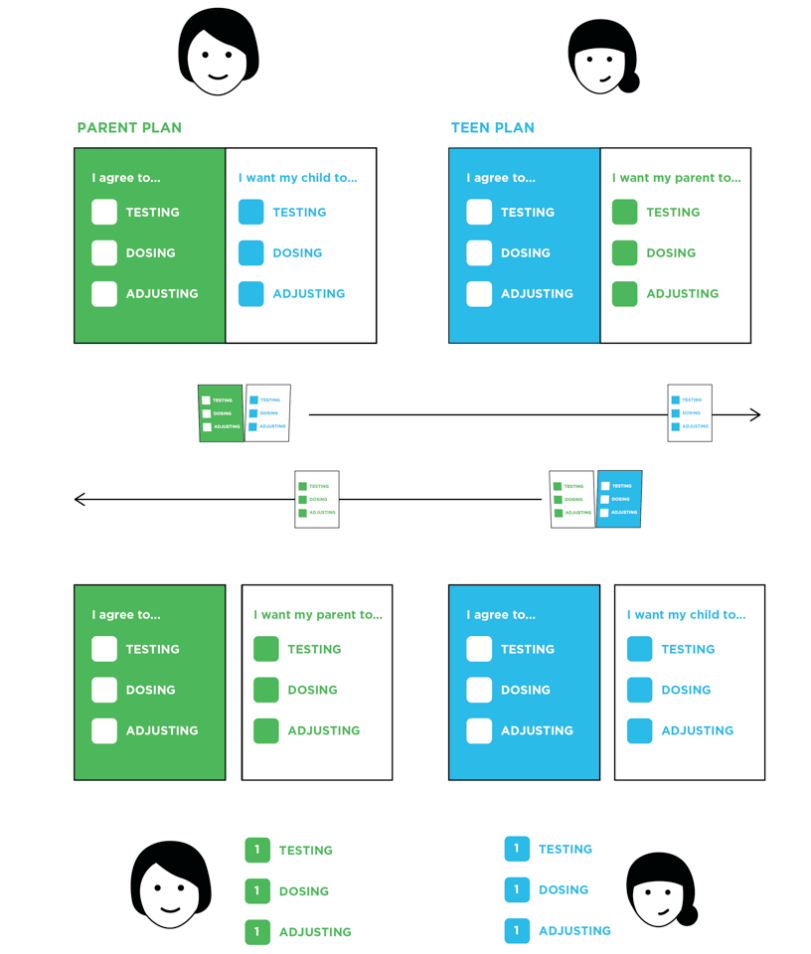
Using the diabetes management plan tool consists of both parent and teen independently choosing action items, sharing these with their partner, and prioritizing and agreeing on issues to discuss with their diabetes care provider.

Figure 1 summarizes the diabetes management plan tool process. Both teens and parents received the tool with instructions to (1) independently choose diabetes self-care tasks (action items) they could do better at from a list of suggestions, or come up with their own; (2) choose or create suggested action items for their partner (teen or parent); (3) exchange their chosen action items with their partner; (4) compare action items with their partner and identify similar, agreed-upon, personal action items based on those they chose and those their partner suggested; (5) prioritize up to 3 action items in terms of how hard they thought the items would be to accomplish and decide whether they could make them goals; and (6) decide on a point tracking system to reward achievement of goals. We focused on 4 aspects of the prototype in this session: functionality and readability, content, use in context, and a reward system.

Multimedia Appendix 1 provides the initial parent and teen versions of the tool from session 2 and Multimedia Appendix 2 shows the final versions designed after the iterative process.

#### Functionality and Readability

Several issues became clear during the session and were later resolved through revisions to the prototype (Multimedia Appendix 1): (1) at least two dyads began by completing step 1 together rather than separately as it was designed, despite having separate tools for parents and teens; (2) it was not clear how to choose or create a goal; (3) some aspects of the tool were hard to read; (4) the arrangement of the steps on the tool was confusing; and (5) how to share the individual tools between parent and teen was confusing.

We addressed these issues through the following revisions: (1) simplifying the steps by combining steps 1 and 2, and visually highlighting this combination using a black box; and (2) improving visual signaling for important tasks such as setting rewards (creating a visual element to highlight “my reward” and “our reward”) and swapping pages (creating a swap symbol and using color in the text to specify which color sheets each partner should have at various steps in the process) (Multimedia Appendix 2).

#### Content

Parents and teens identified 2 specific words within the prototype as problematic: *yelling*, which was a turnoff for parents, and *fasting* (referring to the time period before the breakfast SMBG check), which some found confusing. We removed these in the final prototype.

#### Use in Context

Overall, parents and teens felt that the usefulness of the tool would depend on family dynamics. For example, one parent thought the tool seemed like “a step back” for their family because they already had an unwritten agreement in place that was working for them. Participants agreed that it would be of better use for families having at least some conflict. All parents agreed that it was important to gear a management plan toward improving *medical* outcomes (eg, hemoglobin A_1c_ in target range). The PAB recommended that a health care provider give guidance for establishing appropriate goal action items and making sure they were specific, measurable, achievable, and results focused. These did not affect the tool itself but were important considerations for how the tool should be used.

#### Reward System

In general, parents and teens thought that a reward system for achieving action items would be a helpful motivator. Some parents expressed that they used punishment only when their teen would not comply with their diabetes management and they felt that a reward might be helpful. The concept of a team reward for achieving action items was considered positive overall. However, one parent stated that punishment may be needed if teens didn’t follow through with action items. The PAB recommended to keep both individual and team rewards in the diabetes management plan tool. They thought that some parents were likely to use punishment whether or not the tool included these kinds of consequences for failure to uphold agreement terms. The personal and team reward remained in the final prototype based on this feedback.

**Table 3:** Acceptability of intervention.

Priorities	Incentives and desires	
	Teen	Parent
Comanagement and conflict resolution	Hold parents accountable for behavior, reactions, and projection of worry Better comanagement relationship	Hold teens accountable for self-care tasks Better comanagement relationship
Focus on common goals	Domains of fear and worry, normalcy, and conflict addressed; not just hemoglobin A_1c_	Coparents agree to work together The process is customizable

## Discussion

### Principal Results

Our objective was to codesign with a PAB an intervention that used shared decision making in the creation of a diabetes management plan. The PAB identified 6 principal domains related to diabetes distress that significantly affected their lives on a day-to-day basis. These were stress, fear and worry, mealtime struggles, assumptions and judgments, feeling abnormal, and conflict. These indicators of diabetes distress relate both directly and indirectly to the ability to perform diabetes self-care and achieve glycemic control [4,37-39]. For example, individuals can be knowledgeable and capable but not follow through due to prioritization of issues of greater importance to them at the present time (eg, fitting in with peers). We found that, although participants stated that routinely measured medical outcomes such as hemoglobin A_1c_ were important outcomes, they did not prioritize these over indicators of diabetes distress (domains) listed by these families at this point in their journey with T1DM. Moreover, parents tend to depend on their diabetes care providers to set glycemic goals [40].

The PAB consensus was that 2 objectives were of principle importance when introducing a diabetes management plan for teens: (1) focusing on conflict resolution strategies, and (2) having an agreed-upon, common goal that was documented and discussed during the clinical visit (Table 3). Diabetes-specific family conflict is well known to affect glycemic control in teens with T1DM [8,41]. Research supports ongoing intervention designed to reduce family conflict in order to improve diabetes-related outcomes [8,11,12,16,42]. However, resources to address family conflict, including access to social work services, family counseling, and psychological services, are sparse in clinical diabetes care. The desires of patients to have these services and the evidence that they are related to superior diabetes outcomes should encourage the field to push for integrating them in the diabetes clinic setting.

One of the results of this work was a cocreated diabetes management plan tool for use in the clinic with teens and their parents. This tool aims to assist with patient-centered goal setting and to suggest that families reward themselves for successes with diabetes self-care. We designed the tool to be individualized. However, one teen expressed that the suggested goal behaviors were too easy and unnecessary because he did not have problems with most of the behaviors listed as examples. This indicated that some patients have trouble thinking outside the box or beyond what is written down on handouts. We also meant the tool to encourage positive reinforcement of both teens and parents by both teens and parents via incentives and rewards. There is evidence that incentives or rewards can have a positive impact on SMBG, but not necessarily on glycemic control [43]. Positive feedback can potentially lessen diabetes-specific family conflict though, and this is of great importance to families [44].

Most of the patient-centered diabetes distress domains discussed by the PAB could be measured using previously published questionnaires. The domains least easily measured are assumptions and judgments, and feeling such as being a “normal” teen, which includes advocating for being treated similarly to teens without T1DM and inclusion. These specific domains address whether parents and teens have the skills, confidence, and knowledge to educate themselves about diabetes and advocate for support and acceptance as needed to improve their quality of life. These skills represent self-efficacy, optimism, or resilience, which are more difficult to measure but have been linked with better health outcomes [28].

### Limitations

This work involved a small number of participants who were recruited from a single geographic area, which could affect the generalizability of the findings. Individual responses could have been influenced by social desirability. Members of the PAB were representative of the condition of interest (teens living with T1DM and their parents). Some participants were lost for session 2 due to scheduling difficulties, which likely affected our findings. For example, the teen participants of the second session were all male, and it is possible that boys and girls have different diabetes care priorities. This likely affected the prototyping and development of the diabetes management plan tool, as other participants (or a larger number of participants) may have recommended differing suggestions for the tool. Although the small sample size included in this first project means that our results are not generalizable, the results have direct implications for our future work. As we test and further codevelop the tool with patients, we will want to involve as many participants as possible. It was not our intention to develop a generalizable intervention in this project, but to develop an intervention to be tested in a separate clinical study with significantly more participants.

### Conclusion

Despite these limitations, our study is an important first step to examining patient-centered outcomes among teens with T1DM by demonstrating that patients with T1DM can be effectively engaged and involved in patient-centered research design. This is important for patient-centered outcomes research to help persons with diabetes achieve personal goals and address diabetes distress. Teens with T1DM prioritize reducing family conflict and fitting into their social milieu over health outcomes at this time in their lives. It is important to acknowledge this when designing interventions to improve health outcomes in teens with T1DM.

## Appendix

Multimedia Appendix 1 Diabetes management plan tool prototypes for parents and teens. Iteration 1 of the tool, with comments on changes made during the coparticipatory process.

Multimedia Appendix 2 Diabetes management plan tool final product for parent and teens.

## Abbreviations

HIT: health information technology
PAB: patient and parent advisory board
PEC: Patient Engagement Core
REDCap: Research Electronic Data Capture
SMBG: self-monitoring of blood glucose
T1DM: type 1 diabetes mellitus
